# Screening, assessment and diagnosis in the eating disorders: findings from a rapid review

**DOI:** 10.1186/s40337-022-00597-8

**Published:** 2022-06-07

**Authors:** Emma Bryant, Karen Spielman, Anvi Le, Peta Marks, National Eating Disorder Research Consortium, Phillip Aouad, Sarah Barakat, Robert Boakes, Leah Brennan, Emma Bryant, Susan Byrne, Belinda Caldwell, Shannon Calvert, Bronny Carroll, David Castle, Ian Caterson, Belinda Chelius, Lyn Chiem, Simon Clarke, Janet Conti, Lexi Crouch, Genevieve Dammery, Natasha Dzajkovski, Jasmine Fardouly, John Feneley, Nasim Foroughi, Mathew Fuller-Tyszkiewicz, Anthea Fursland, Veronica Gonzalez-Arce, Bethanie Gouldthorp, Kelly Griffin, Scott Griffiths, Ashlea Hambleton, Amy Hannigan, Mel Hart, Susan Hart, Phillipa Hay, Ian Hickie, Francis Kay-Lambkin, Ross King, Michael Kohn, Eyza Koreshe, Isabel Krug, Anvi Le, Jake Linardon, Randall Long, Amanda Long, Sloane Madden, Sarah Maguire, Danielle Maloney, Peta Marks, Sian McLean, Thy Meddick, Jane Miskovic-Wheatley, Deborah Mitchison, Richard O’Kearney, Roger Paterson, Susan Paxton, Melissa Pehlivan, Genevieve Pepin, Andrea Phillipou, Judith Piccone, Rebecca Pinkus, Bronwyn Raykos, Paul Rhodes, Elizabeth Rieger, Sarah Rodan, Karen Rockett, Janice Russell, Haley Russell, Fiona Salter, Susan Sawyer, Beth Shelton, Urvashnee Singh, Sophie Smith, Evelyn Smith, Karen Spielman, Sarah Squire, Juliette Thomson, Marika Tiggemann, Stephen Touyz, Ranjani Utpala, Lenny Vartanian, Andrew Wallis, Warren Ward, Sarah Wells, Eleanor Wertheim, Simon Wilksch, Michelle Williams, Stephen Touyz, Sarah Maguire

**Affiliations:** 1 InsideOut Institute for Eating Disorders, Faculty of Medicine and Health, University of Sydney, Sydney, NSW Australia; 2 Sydney Local Health District, New South Wales Health, Sydney, Australia; 3 Healthcare Management Advisors, Melbourne, VIC Australia

**Keywords:** Screening, Diagnosis, Assessment, Eating disorders, DSM-5, Early intervention, Nosology, Psychometrics

## Abstract

**Background:**

Limited screening practices, minimal eating disorder training in the healthcare professions, and barriers related to help-seeking contribute to persistent low rates of eating disorder detection, significant unmet treatment need, and appreciable associated disease burden. The current review sought to broadly summarise the literature and identify gaps relating to the screening, assessment, and diagnosis of eating disorders within Western healthcare systems.

**Methods:**

This paper forms part of a Rapid Review series scoping the evidence base for the field of eating disorders, conducted to inform the Australian National Eating Disorders Research and Translation Strategy 2021–2031, funded and released by the Australian Government. ScienceDirect, PubMed and Ovid/Medline were searched for studies published between 2009 and mid 2021 in English. High-level evidence such as meta-analyses, large population studies and Randomised Control Trials were prioritised through purposive sampling. Data from selected studies relating to Screening, Assessment and Diagnosis of eating disorders were synthesised and are disseminated in the current review.

**Results:**

Eighty seven studies were identified, 38% relating to screening and 62% to assessment and diagnosis. The majority of screening studies were conducted in university student samples, showing high prevalence but only modest improvements in help-seeking in those studies that followed up post-screen. In healthcare settings, clinicians continue to have difficulty identifying eating disorder presentations, particularly Binge Eating Disorder, Other Specified Feeding or Eating Disorders, and sub-threshold eating disorders. This is preceded by inadequate and frequently homogenous screening mechanisms and exacerbated by considerable personal and health-system barriers, including self-stigma and lack of resourcing. While all groups are at risk of delayed or no diagnosis, those at particular risk include LGBTQ+ and gender diverse individuals, individuals living in larger bodies, and males.

**Conclusions:**

A majority of individuals with eating disorders remain undiagnosed and untreated despite a high prevalence of these conditions and increased advocacy in recent years. Research into improving detection and clinician diagnostic skill is extremely limited. Innovative empirical research is strongly recommended to address significant individual and health-system barriers currently preventing appropriate and timely intervention for many.

**Plain English Summary:**

Limited screening in healthcare settings and low rates of eating disorder training in the healthcare professions are just some of the barriers to help-seeking which may contribute to delayed intervention and diagnosis in the eating disorders. This has significant impacts, prolonging treatment when it is finally received, and increasing healthcare costs for both the individual and the healthcare system. The current review is part of a larger Rapid Review series conducted to inform the development of Australia’s National Eating Disorders Research and Translation Strategy 2021–2031. A Rapid Review is designed to comprehensively summarise a body of literature in a short timeframe, often to guide policy-making and address urgent health concerns. The Rapid Review synthesises the current evidence-base and identifies gaps in eating disorder research and care, in order to guide decision making and address urgent health concerns. This paper gives a critical overview of the scientific literature relating to the current state of screening, assessment, and diagnosis of eating disorders within Western healthcare systems that may inform health policy and research in an Australian context. It covers screening initiatives in both general and high-risk populations; personal, clinician and healthcare system challenges relating to help-seeking; and obstacles to accurate and timely clinical diagnosis across the eating disorders.

**Supplementary Information:**

The online version contains supplementary material available at 10.1186/s40337-022-00597-8.

## Introduction

Eating disorders (EDs) are complex neuropsychiatric conditions from which individuals can fully recover, however recovery remains elusive for many. Approximately 50% of individuals fully recover, taking on average 1–6 years to achieve [1–4], while 20–30% develop a chronic course [4–6]. Delays in intervention, where treatment does not occur until illness behaviours and cognitions are entrenched, contribute to a protracted illness course [7, 8]. As with any mental illness, early intervention—predicated on timely screening, assessment and diagnosis—positively impacts prognostic outcome and overall disease burden [7, 9–11]. However, despite significant disability and mortality, low rates of ED screening at an individual and population level, perceived stigma and personal reluctance to seek care, and lack of early identification in primary care means opportunities to intervene early are frequently missed [12–14].

Primary healthcare personnel typically have minimal training in EDs and have difficulty identifying, diagnosing or managing these conditions [12, 14, 15]. Despite high rates of disordered eating behaviour in the Australian general population [16–18], including between 31.6 and 51.7% of adolescents [19–21], there is evidence to suggest both disordered eating and full syndrome EDs are being underdiagnosed in primary care settings [22, 23]. Clinicians report greater awareness of diagnostic criteria for Anorexia Nervosa (AN) and Bulimia Nervosa (BN), compared to other ED diagnoses; in particular Binge Eating Disorder (BED) and Other Specified Feeding or Eating Disorders (OSFED) (including Atypical AN (A-AN)) [23–25]. Improving clinical knowledge of diverse ED behaviors and symptoms will be important for ensuring accurate diagnosis and assessment of EDs in primary care settings and enabling early intervention in the form of timely referral and access to appropriate care per diagnostic type [22]. Further, understanding the state of assessment and diagnosis and its influence on patient-care pathways will be critical to clinical education and resourcing.

Limited screening practices and tools within both healthcare and more diverse settings is an additional barrier to early intervention for individuals impacted by EDs, and means even high-risk groups such as dieting adolescents, women seeking reproductive healthcare, and individuals with diabetes and other comorbidities are not screened, and early symptomatology undetected [26, 27]. Introducing both targeted and population health strategies such as ED education programs or population screening in schools, and utilising web-based technologies to encourage individuals to seek support may drive increased identification, which can lead to early intervention [28, 29].

The current Rapid Review (RR) paper is one of a series scoping the field of EDs, commissioned by the Australian Federal Government to inform the Australian National Eating Disorders Research and Translation Strategy 2021–2031 [30]. This review seeks to identify and summarise population-based screening approaches and evidence relevant to the clinical assessment and diagnosis of EDs to date, with a focus on Western healthcare systems that may inform health policy and translational research in an Australian context.

## Methods

The Australian Government funded the InsideOut Institute for Eating Disorders (IOI) to develop the Australian Eating Disorders Research and Translation Strategy 2021–2031 [30] in partnership with state and national stakeholders including clinicians, service providers, researchers, and experts by lived experience (including consumers and families/carers). Developed through a two-year national consultation and collaboration process, the strategy provides the roadmap to establishing EDs as a national research priority and is the first disorder-specific strategy to be developed in consultation with the National Mental Health Commission. To inform the strategy, IOI commissioned Healthcare Management Advisors (HMA) to conduct a series of RRs to broadly assess all available peer-reviewed literature on the six DSM-5 listed EDs.

A RR Protocol [31] was utilised to swiftly synthesise evidence in order to guide public policy and decision-making [32]. This approach has been adopted by several leading health organisations including the World Health Organisation [33] and the Canadian Agency for Drugs and Technologies in Health Rapid Response Service [34], to build a strong evidence base in a timely and accelerated manner, without compromising quality. A RR is not designed to be as comprehensive as a systematic review—it is purposive rather than exhaustive and provides actionable evidence to guide health policy [35].

The RR is a narrative synthesis and sought to adhere to the PRISMA guidelines [36]. It is divided by topic area and presented as a series of papers. Three research databases were searched: ScienceDirect, PubMed and Ovid/Medline. To establish a broad understanding of the progress made in the field of eating disorders, and to capture the largest evidence base from the past 12.5 years (originally 2009–2019, but expanded to include the preceding 1.5 years), the eligibility criteria for included studies into the rapid review were kept broad. Therefore, included studies were published between 2009 and mid 2021, in English, and conducted within Western healthcare systems or health systems comparable to Australia in terms of structure and resourcing. The initial search and review process was conducted between 5 December 2019 and 16 January 2020. Initial screening of articles based on their titles/abstracts was conducted by three independent HMA reviewers (led by AL) as part of the search strategy process. Articles assessed for inclusion underwent a further review process based on the evidence presented with relevance for the RR—this was conducted by two HMA reviewers (AL+ colleague) involved in initial screening. The re-run for the years 2020–2021 was conducted by two reviewers (EB+ colleague) on the 30th May 2021, adopting the same review process. Disagreement on studies for inclusion/exclusion in both instances was resolved by discussion between the reviewers with disputes referred to an expert research panel for final decision. Evidence presented in the RR is based on literature that satisfied criteria following this subsequent review process.

The RR had a translational research focus with the objective of identifying evidence relevant to developing optimal care pathways. Searches therefore used a Population, Intervention, Comparison, Outcome (PICO) approach to identify literature relating to population impact, prevention and early intervention, treatment, and long-term outcomes. Purposive sampling focused on high-level evidence studies such as: meta-analyses; systematic reviews; moderately sized randomised controlled studies (RCTs) (*n* > 50); moderately sized controlled-cohort studies (*n* > 50), or population studies (*n* > 500). However, the diagnoses ARFID and UFED necessitated a less stringent eligibility criterion due to a paucity of published articles. As these diagnoses are newly captured in the DSM-5 (released in 2013, within the allocated search timeframe), the evidence base is emerging and fewer studies have been conducted. Thus, smaller studies (n = < 20) and narrative reviews were also considered and included. Grey literature, such as clinical or practice guidelines, protocol papers (without results) and Masters’ theses or dissertations, was excluded. Instrument validation studies and studies commenting on the current *Diagnostic and Statistical Manual of Mental Disorders* (*DSM-5*) criteria for EDs were also excluded as they were not seen to be relevant to the patient-care focus of the review.

Full methodological details including eligibility criteria, search strategy and terms and data analysis are published in a separate protocol paper due to the broad scope of the RR, which included a total of 1320 studies [37] (see Additional File 1 for PRISMA flow diagram). Data from included studies relating to Screening, Assessment and Diagnosis were synthesised and are presented in the current review. No further analysis was carried out on reported results.

## Results

The RR identified 87 studies for inclusion in the ‘Screening, Assessment and Diagnosis’ category. Approximately 38% (*n* = 33) related to screening and 62% (*n* = 54) to assessment/diagnosis, with a significant number of studies conducted in university settings (*n* = 8), reproductive healthcare settings (*n* = 12), and with children (*n* = 12). Given the scale of the RR, there is a demonstrable lack of evidence on screening, assessment, and diagnosis which made up 6% of the total body of literature reviewed for the full series. A full list of included studies for this topic, including population, aims, and outcome measures can be found in Additional File 2. Results are subdivided into two categories: (i) screening; and (ii) assessment and diagnosis.

## Screening

Studies identified by the RR typically assessed the efficacy and scalability of screening programs within particular settings, frequently capturing high-risk groups such as students, and individuals presenting to healthcare services with related physical or mental health concerns.

### University and online screening programs

Recognising the higher risk and elevated prevalence of EDs among adolescents and young adults [38], numerous screening studies have been conducted in university student samples and online, where many young people seek initial healthcare information [39].

In a US-based sample of undergraduate and postgraduate students, 9–13% of female students screened positive for an ED, while among males it was 3–4% [40, 41]. Further assessment of this student population indicated that among those screening positive, very few had received a diagnosis or sought help for their ED. The authors suggested this pointed to a lack of urgency or importance placed on ED symptomatology in this group, identifying a need for university-based interventions and large screening programs in this high-risk population [40].

In a university-based screening program conducted in France, high rates of EDs (up to 25%) were detected among 1493 female and male students and were associated with increased substance use and cyber-addiction [42]. Tavolacci et al. [42] suggested that all students should be screened for an ED upon admission to university and referred to a general practitioner if required, also noting a need for the delivery of ED prevention programs in university settings. Researchers identified a high number of male students (10%) showing ED symptomatology, requiring greater attention within prevention and treatment efforts [42]. Male university students were also found to have limited health literacy concerning eating disorders, perceiving eating disorders to be less severe and less distressing conditions than did their female counterparts [43]. Authors described a particular need to target the attitudes and beliefs of young males in prevention and early intervention initiatives.

A substantial amount of research undertaken in university student samples has occurred through the US ‘Healthy Body Image’ (HBI) program, which targets undergraduate students with the objective of identifying those at high risk of EDs and linking them into prevention or treatment interventions [28]. Delivered as an online screener, participants are offered prevention programs or further evaluation based on their assessed level of ED risk. Three levels of cognitive-behavioural-based online interventions are offered to participants to encourage healthy weight and prevent ED onset in high-risk students [44]. Fitzsimmons-Craft et al. [28] noted considerable uptake in these interventions when assessing three years of state-wide deployment of HBI in Missouri public universities: between 44 and 51% of individuals screened. Participation was particularly high among older students over the age of 25, which was suggested to be a benefit of the convenience of access afforded by online interventions [28].

Despite a high level of engagement in the research, lack of a perceived need for treatment among US college students appears to be a significant issue. Seeking to identify barriers to help-seeking among students screened for the HBI program, Lipson et al. [29] reported that a high proportion (41.7%) of individuals who exhibited ED symptomatology indicated that they ‘have not had a need for counselling/therapy’ [29]. Almost one-fifth (19.9%) were unsure of how serious their needs were or ‘[didn’t] have time’ (19.5%) to address their symptoms. Only a small proportion of participants raised personal stigma as a barrier to help-seeking (4.1%), with low levels of stigma reported by the cohort overall [29]. Following this initial rollout, the HBI program expanded to 28 sites across the US. Overall, it demonstrated scalability of online programs and their capacity to identify high-risk individuals who may not naturally seek help, whilst referring them to effective online interventions [45].

Further expansion of online ED screening in 2017 by the United States’ National Eating Disorders Association (NEDA) website found high levels of unmet treatment need in the 71,362 members of the general public who were screened [46]. It was unclear whether participants in this screening program were offered the same interventions offered to those in the HBI program. In the 18 months to August 2019, the NEDA website screen identified over 340,000 people at high risk or symptomatic of ED, out of a total 353,115 completers [47]. Researchers sought to understand help-seeking intentions of these individuals post-screen completion. Only 4.8% of eligible respondents provided this data and of those, just one-third expressed help-seeking intentions, with 16% initiating treatment [47], suggesting online screening initiatives should consider ways to increase motivation for help-seeking and treatment-uptake.

Fitzsimmons-craft et al. [48] conducted a 9 months follow up of individuals who had screened positive for AN as part of the HBI program. Again, only 26% initiated treatment (however, 33% reported already being in treatment at the time of the screen). Participants reported feeling ashamed, nervous, and sad, but also validated, in response to the positive screen. The strongest barriers to treatment included believing one should be able to help themselves, believing the problem was not serious enough to warrant treatment, and being time-poor [48].

Poor uptake of treatment post-screening is not limited to online screening programs. Screening of EDs among individuals attending a smoking cessation program also highlighted issues relating to the reluctance to seek help among patients even after assessment. Only 17% of the population assessed as having an ED accepted a referral to a specialist ED clinic [49]. Thus, while large scale screening programs may help individuals identify their ED, careful consideration should be given to delivering screening programs without proper linkage to effective and appropriate interventions, especially considering the low tendency toward help-seeking among individuals with or at high-risk of an ED [40, 46].

Such linkage between a large online screening program and prevention or treatment interventions was effectively demonstrated by the ProYouth initiative delivered across seven European countries (Germany, Ireland, Czech Republic, Romania, the Netherlands, Italy, and Hungary). Following completion of an online screening tool available through the website, participants could access psychoeducation on EDs, peer or professional support through moderated online forums, or were provided details of available qualified professionals or specialist ED services in their area based on their level of need. Szabo et al. [50] noted that while the screening program addressed some unmet need in the population, lack of specialist ED services may present a barrier to individuals with the most severe ED symptoms.

One screening program specifically targeting males was identified by the RR. Consistent with evidence from screening interventions discussed previously, results from the screening program of 16–20-year-old males indicated that ED behaviours were a significant issue among both sexes. Domine et al. [51] argue that more screening interventions must be undertaken in male populations.

### Primary care and specialist healthcare settings

Evidence indicates there is clinical utility in undertaking screening for EDs in primary care settings [52]. The capacity of existing screening tools to capture all DSM-5 EDs in this setting has been questioned by researchers who indicate potential cases may be over- or under-diagnosed [52]. The RR does not seek to comment on the validation of specific screening tools; rather, it discusses the importance of screening for EDs in groups where there is a need for clinicians involved in the care of concomitant health issues to understand elevated risk.

One such group is individuals experiencing rapid and significant weight gain presenting to primary care, where research demonstrates screening for BED should occur. In a study screening for BED in this population, those who screened positive had gained on average 8.2 kg in one year, while those who did not had gained an average of 0.7 kg [53]. Research conducted in samples of college students in the US lends further support to this argument. Kass et al. [54] found in their study evaluating differential ED screening results by weight range in a sample of university students, that within the group of students in the overweight/obese weight range, 58% were identified as high-risk or warranted clinical referral [54]. Compared to individuals in the normal and underweight ranges, this group was more likely to endorse objective binge eating and fasting, ED-related concerns that impaired their psychosocial functioning, and higher weight/shape concerns. Similarly, a 2018 Australian study found that in the general population, strict dieting was associated with elevated Body Mass Index (BMI) (and not with low BMI as might be expected) [22]. Individuals of elevated or increasing BMIs should be routinely screened for ED psychopathology.

Evidence also suggests increased efforts should be made to screen for EDs among individuals with type 1 diabetes. Generally required to undertake dietary restriction to manage their condition, research indicates that disordered eating is elevated in this group (affecting between 10 and 39%) [26, 55]. Hanlan et al. [26] noted that ED screening tools developed for the general population may produce false positive results in people with type 1 diabetes due to the necessity for these individuals to monitor diet in a way that might be seen as pathological in the general population. Some diabetes-specific screening tools exist and are effective for adults (e.g., the Diabetes Eating Problem Survey (DEPS)), however evidence on the effectiveness of current screening for EDs in adolescents with diabetes was less conclusive [26]. Eilander et al. [56] suggested a pertinent ‘yellow flag’ that may indicate ED risk among diabetic adolescents is shape and weight concern, and that this should be routinely screened for. Findings emphasise the need to increase clinician awareness and related screening in all healthcare settings considering the high-risk status of adolescents and increased morbidity and mortality associated with the co-occurrence of EDs and type 1 diabetes [26].

In the US, provision of an in-person and online education program delivered to over 300 primary care clinicians demonstrated effectiveness in their capacity to carry out screening for EDs in paediatric patients, with in-person shared learning followed by consistent online education demonstrating superior efficacy over the provision of printed materials [15]. Research into whether such a program delivered to Australian clinicians working across high-risk settings could effectively improve screening and consideration of how participation in this professional development may be incentivised to ensure uptake may be warranted.

### Mental health and specialist psychiatric services

Despite EDs being commonly associated with mental health comorbidities, only two studies looking at screening in general mental health or specialist psychiatric services were identified. Lobera et al. [57] argued that EDs are rarely assessed among patients seeking treatment for other psychiatric conditions in their study, which reported increased bulimic symptoms among patients with anxiety and depressive disorders that had not been identified in routine mental health care. A second study comparing women accessing psychiatric inpatient care to those using primary and obstetric services in Australia, found ED symptomatology was significantly higher among the psychiatric inpatient group [58], indicating a need for clinicians providing psychiatric care to be aware of and screen for EDs that may negatively impact their psychiatric outcomes. Given the elevated rates of EDs among individuals seeking mental health and psychiatric services, researchers have suggested screening for EDs should be built into routine assessments to identify treatment need [59].

### General hospitals

Only one study was identified examining the need for ED screening of patients in a general hospital setting. Detected prevalence of EDs among patients seeking emergency department services in the US was 16% and did not differ by reason for seeking treatment or any socio-demographic factors. Dooley-Hash et al. [60] indicated this was much higher than the estimated prevalence in the general population (5%) and suggested that emergency department presentation could represent an important step in the identification of patients with ED for referral to services [60].

### Women’s reproductive health services

A considerable amount of evidence on ED screening relates to women seeking treatment for infertility and gynaecological care due to the known negative impact of EDs on reproductive health [61, 62].

There is substantial evidence of heightened ED risk among women with polycystic ovarian syndrome (PCOS). In a systematic review of 21 studies, Paganini et al. [63] identified a shared risk factor between PCOS and binge/purge type disorders precipitated by body dissatisfaction. Among Australian women aged between 22 and 27, individuals with PCOS had higher rates of EDs (11% compared with 7.6%), lower self-esteem, and higher rates of psychological distress than women without PCOS [64]. Detected prevalence of EDs among women in the obese weight range with PCOS may be even higher in a study of women in the UK, 39% of women in the obese weight range with PCOS exhibited clinically significant binge eating behaviour [65]. Further, a meta-analysis (Lee et al. [66]) of seven studies showed risk of ED among women with PCOS was three times higher than in healthy women. However, this figure was slightly lower in a 2019 study of Australian women by Tay et al. [64], who found one and a half times the risk of EDs (other than AN and BN) in those women with PCOS.

In two US studies, Cousins et al. [61] and Freizinger et al. [67] emphasised a need for screening among women with unexplained infertility, as they were more likely to display drive for thinness, bulimic symptoms, and past history of undisclosed infertility. Contrarily, assessment of ED symptomatology among Australian women seeking treatment for infertility did not indicate that rates were higher in this group than in the general population [68]. The discrepant outcomes may be a reflection of the different constructs used to assess for eating disorder symptomatology. The former study employed the Eating Disorder Inventory-3 (EDI-3) (Garner 2004) and reported significantly higher scores on that instrument’s ‘drive for thinness’ and ‘bulimic symptom’ subscales for individuals with unexplained infertility compared to those without. The latter used the Eating Disorder Examination Questionnaire (EDE-Q) (Fairburn and Beglin 2006), which lends its focus to overvaluation of weight and shape and does not include bulimic behavioural symptomatology in its global score.

A study on the knowledge, attitudes, and clinical practices of fertility specialists in Australia and New Zealand indicated that, while clinicians consider screening for EDs in this context to be important due to the identified association between inadequate nutrient intake and development of risky pregnancy, they experienced a significant amount of uncertainty as to what actions should be taken following assessment [69–71]. These issues were also common to clinicians in the UK where a study identified lack of knowledge and uncertainty of clinician role to be barriers to the identification of EDs among pregnant and postnatal women, alongside issues such as stigma and taboo [72]. Authors suggested a need for better health professional education and development of guidelines to ensure high-quality routine care for women with EDs in preconception and prenatal care [70, 72].

### Bariatric surgery services

Due to frequent high body weight [73] individuals with binge eating pathology may seek out bariatric surgery for weight reduction [74, 75]. Ten studies identified increased risk of ED in this population. Prevalence of BED in candidates for bariatric surgery has been reported up to 45%, prevalence of NES up to 59%, OSFED up to 32% and BN up to 2% [74, 76–81]. Conversely, in an adolescent sample of bariatric patients, prevalence rates were much lower at 7% for BED and 5% for NES [80]. Several studies have noted that a diagnosis of BED is a known predictor of poor weight loss and continued poor quality of life following bariatric surgery [80, 82, 83]. Additionally, research has explored the impact of delivering psychological and behavioural interventions to patients with EDs prior to surgery, showing the provision of CBT to patients with BED before their surgery improved surgery outcomes and resulted in a longer-term reduction of binge eating and weight loss. Therefore, screening for eating disorders in candidates for bariatric surgery is strongly indicated for the improvement of both physical and psychological outcomes [74, 84].

### ARFID

Limited evidence was found for screening or identifying Avoidant Restrictive Food Intake Disorder (ARFID) (*n* = 2). Burton Murray et al. [85] surveyed adult patients referred for gastroparesis/dyspepsia symptoms at two academic medical centres in 2018/2019 for gastrointestinal symptom severity and broad Feeding or Eating Disorder (FED) symptoms. FED symptoms were associated with greater gastrointestinal symptom severity, but not gastric retention [85]. The authors suggested that while clinicians should be cautious about diagnosing ARFID in gastroparesis/dyspepsia patients, screening for the condition may assist in the establishment of appropriate referral pathways.

A 2020 systematic review of the literature identified just 5 of 77 total ARFID articles were related to screening, diagnosis, or assessment. Two articles examined tools to generate a diagnosis of ARFID: the Pica, ARFID and Rumination Disorder Interview (PARDI) and the Eating Disorder Examination—ARFID module (EDE-ARFID). Both showed good psychometric properties, though were validated in small sample sizes [86]. Three articles presented empirical data on screening instruments designed to identify ARFID: the Eating Disturbances in Youth Questionnaire (EDY-Q; Hilbert and van Dyck, 2016) and the Nine Item ARFID Screen (NIAS) (Zickgraf and Ellis 2018) [87–89]. Both showed promising results with further study warranted, noting that the literature regarding ARFID screening, particularly in adults, is scant [86].

### Other high-risk populations

Data on screening transgender and gender diverse populations for ED symptomatology is also limited, despite their elevated risk for the condition [90, 91]. One study aimed to understand transgender and gender diverse young adults’ experiences of ED screening and treatment using qualitative methodology. Sixty-six participants aged 18–30 raised three major themes: barriers to ED screening/treatment; complexity of the relationship between EDs and gender dysphoria; and need for provider education in gender affirming care practices for ED screening and treatment. Twenty-eight percent of participants identified barriers to ED screening and treatment [92]. These included structural barriers, ED stereotypes (i.e., that they only affect cis gender, white females) undermining identification and treatment access, and a discordance between traditional ED treatments and gender-affirming practice (for example, body acceptance, which is seen as a vital component of the former but not the latter). Lack of affordability and a paucity of mental health providers were also identified barriers to treatment [92].

A study using data from the NEDA online screening tool compared ED treatment seeking behaviours of self-identified competitive athletes and non-athletes during the 2018 NEDA Awareness Week. Over 86% of the 23,000 respondents met criteria for an ED/subthreshold ED, and only 2.5% were in treatment. 14.7% of the total sample identified as being a competitive athlete [93]. Athletes were more likely than non-athletes to screen positive for an ED, but there was no significant difference when it came to treatment history or intention to seek treatment. Less than 30% of all individuals screening positive intended to seek treatment post-screen [93].

It has been suggested that children of mothers with a history of EDs should also be regularly screened. Within a large cohort of mothers and female children, lifetime EDs in mothers was associated with a greater risk of daughters developing ED symptomatology and higher likelihood of presentation to ED treatment [94]. Screening both mothers and daughters for current or previous disordered eating may be important for the prevention and early identification of ED symptoms.

## Assessment and diagnosis

Studies identified by the RR relating to the assessment and diagnosis of EDs investigated rates of help-seeking in the general population, clinician role and skill in the diagnostic assessment of EDs, clinical characteristics (particularly within sub-populations), and diagnostic heterogeneity.

### Help-seeking

A considerable proportion of individuals meeting diagnostic criteria for an ED or displaying problematic disordered eating behaviours do not seek treatment [23, 95]. One systematic review of 14 studies reported a pooled prevalence of treatment seeking of just 23.2%, with many individuals more likely to receive treatment for weight loss than for an eating problem [13]. This unmet health need may be partially mitigated by increasing clinician awareness and assessment of individuals in primary care, subsequently referring them to ED services.

Using data from a national survey sample in the US, it was estimated that only 32% of individuals with AN had ever received treatment for their disorder [96]. For other ED diagnoses, there was a significant difference in treatment provision between genders (estimated 47% of women with BN received treatment, while the proportion in men was 29%). For BED the difference was slightly larger with 49% of women receiving treatment compared with 28% of all men [96]. There is evidence to suggest adolescents are less likely than adults to seek treatment for their ED, notwithstanding this period being a critical age of onset and outcome worsening the longer the duration of illness [17, 97, 98]. Despite comparable prevalence, individuals of ethnic minority are even less likely to receive a referral for their ED than are Caucasian individuals, regardless of age. [99].

Bode et al. [100] estimated that the annual cost to the health system in Germany of untreated AN was €2.4 billion (A$3.7 billion), while for BN the estimation was €617.7 million (A$949 million). Thus, delays in treatment seeking come at a significant cost to the patient as well as the health system.

### Role of the primary healthcare professional

Primary health providers play a pivotal role in the early identification and diagnosis of EDs in the community, providing affected individuals with timely access to care and ideally early intervention [101, 102]. However, in Australia, a review of ED service referrals from primary care practitioners suggested there is lack of awareness regarding signs and symptoms of EDs other than AN and BN [23, 103]. A survey of 136 clinicians working in regional Queensland found that 73% had little or no confidence in working with EDs [104]. An observational study conducted by Allen et al. [23] found primary care practitioners in Western Australia were able to accurately assess and refer patients with AN and BN to specialist services but tended to diagnose EDNOS as AN-like or BN-like disorders [23]. As the specialist service did not provide treatment for BED, lack of referrals received was not considered a gap by Allen et al. [23]. However, evidence from a literature review assessing diagnosis of BED in primary care settings suggested the diagnosis is consistently under-recognised and left untreated despite its high prevalence in the community [105]. In individuals with BED, overvaluation of weight/shape has been identified as a key distinguishing feature (present in an estimated 60% of cases) compared to higher weight individuals without BED. Amianto et al. [106] suggest screening for this core ED symptom in primary care settings may represent an opportunity to provide greater access to care for individuals with BED.

### Diagnostic heterogeneity

A high degree of overlap in the symptomatology of different ED subtypes adds to the challenge of accurately assessing and diagnosing different EDs. For example, an assessment of the clinical characteristics of a sample of women presenting to ED services in Sweden found individuals with AN-BP, BN and Purging Disorder (PD) to have more similarities than differences, lending support to the argument that binge/purge ED subtypes share key features and behaviours that lead to disease onset and maintenance [107]. Ekeroth et al. [107] also pointed to the considerable overlap between diagnostic criteria in Purging Disorder and A-AN as an issue causing diagnostic confusion for clinicians.

In support of the finding from Allen et al. [23] that primary care clinicians may not be accurately diagnosing Eating Disorder Not Otherwise Specified (EDNOS)/Other Specified Feeding or Eating Disorder (OSFED)/Unspecified Feeding or Eating Disorder (UFED), Wade and O’Shea (2014) found that individuals with UFED presenting to services tended to be overweight and therefore may not be assessed as having an ED by clinicians, despite having the same EDE scores as patients with other full threshold disorders [23, 103]. An assessment of UFED symptomatology in a sample of Australian female adolescents by Wade and O’Shea (2014) found that individuals with UFED suffered significant psychological distress and displayed core ED symptomatology including overvaluation of weight/shape at similar levels of severity to women with full threshold EDs [103].

Further, a study utilising the *DSM-5* criteria on an adult patient sample at a specialist ED clinic in Melbourne (Australia) found similar levels of psychiatric comorbidities and symptomatic severity between individuals with OSFED/UFED and those with full threshold disorders [108]. In contrast, findings from the clinical sample of Swedish women discussed in Ekeroth et al. [107] found individuals diagnosed with UFED exhibited significantly less severe symptomatology.

**Fig. 1:**
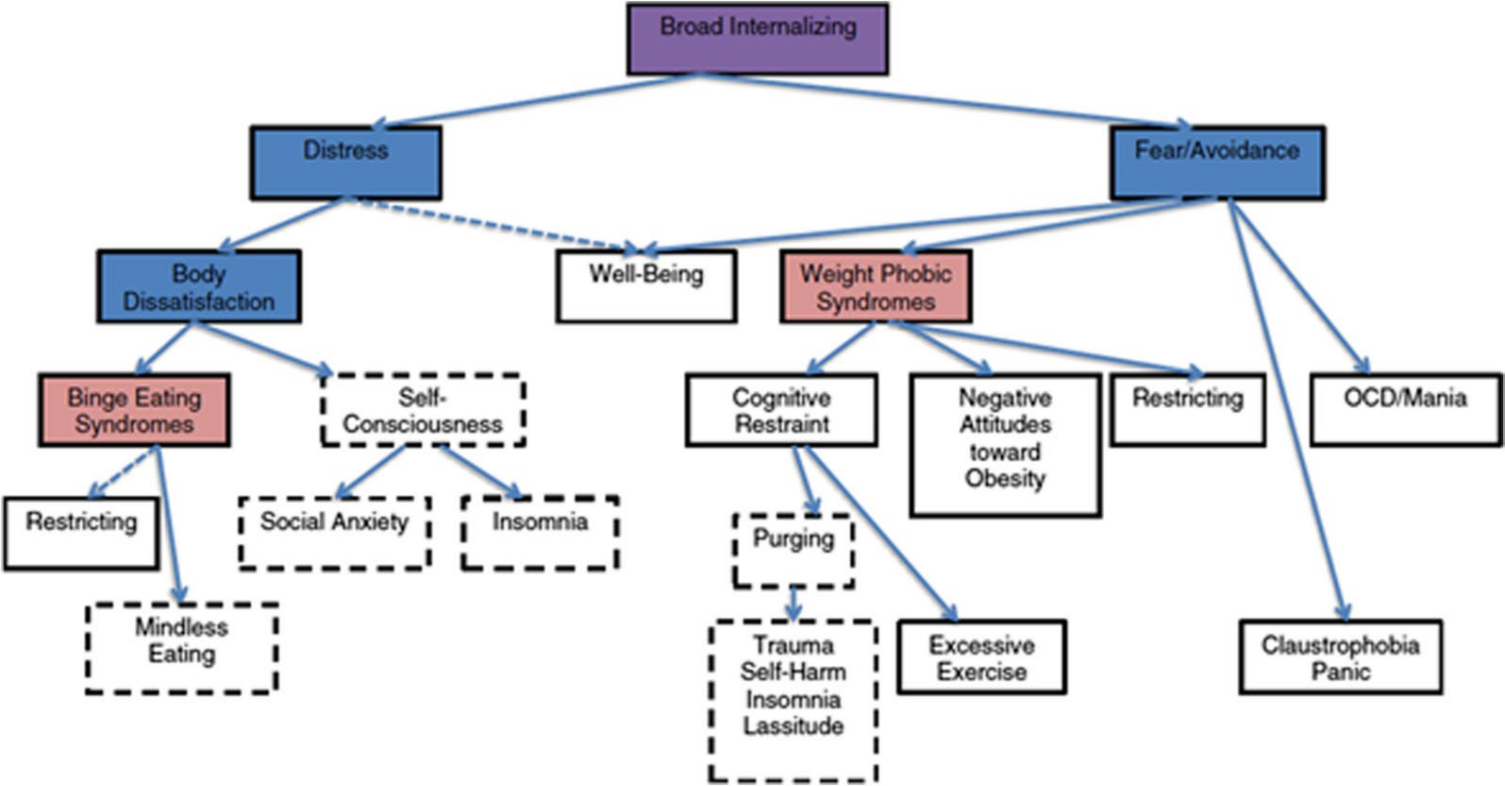
Forbush et al. [110] proposed hierarchical transdiagnostic model of EDs: summary of hierarchical structural associations based on bass-ackwards analysis. Purple boxes represent spectra. Blue boxes represent sub-factors. Pink boxes represent syndromes. Boxes that are not shaded represent homogeneous symptom components. Dashed blue lines are negative correlations. Dashed boxes represent symptom components that formed a “borderline” syndrome that began to emerge at Level 13 of the hierarchical analysis. Forbush et al. [110] (permission to reproduce)

Transdiagnostic models of ED highlight common internalising psychopathologies as central and thus seek to reduce diagnostic heterogeneity, aiming to characterise indicators that are consistent across disorders [109]. A longitudinal community-based cohort study in the US found 15 transdiagnostic factors: (1) Distress, (2) Well Being, (3) OCD and Mania (Thought Disorder), (4) Restricting, (5) Negative Attitudes toward Obesity, (6) Excessive Exercise, (7) Binge Eating, (8) Body Dissatisfaction, (9) Insomnia, (10) Lassitude, (11) PTSD, (12) Social Anxiety, (13) Mindlessness, (14) Purging (self-induced vomiting, and diuretic and laxative misuse), and (15) Claustrophobia, arranging them into a hierarchy that allowed prediction of disorder severity. A summary of their hierarchical structural associations is demonstrated in Fig. 1. The authors argued that clinicians without specialist knowledge of EDs may derive benefit from such a model, allowing for accurate impairment assessment without requiring a detailed knowledge of diagnostic criteria [109].

### Children and adolescents

Issues relating to accurate assessment and diagnosis are particularly apparent in children and adolescents. Eating pathology and behavioural symptoms in children and adolescents at presentation appear to be less pronounced than in adults [110, 111].

Two studies identified by the RR were conducted within specialist paediatric ED services in Western Australia with the aim of examining the applicability of the *DSM-5* criteria in the child and adolescent population, while determining differences in clinical presentation between these subgroups. Walker et al. [110] in an observational study of 656 children and adolescents found distinct differences in presentations between children (aged below 13) and adolescents (aged 13–18) with EDs. While adolescents were more likely to present with binge/purge behaviour and BN-type EDs, children had lower eating pathology, and were less likely to engage in bingeing or compensatory behaviours [110]. However, children were found to lose weight at a much faster rate than adolescents. In addition, a higher relative proportion of children presenting to the service were male (17.3%) than in the adolescent group (4.3%) [110].

Analysis of clinical presentations of EDs among children and adolescents by Swenne [112] indicated that those with high pre-morbid BMIs were in danger of going undiagnosed despite significant weight loss, indicating a need for clinicians to more closely monitor patients with suspected atypical presentations, including A-AN [112]. Similarly, Whitelaw et al. (2014) in their 6-year retrospective cohort study of 12–19-year-old patients at a tertiary children’s hospital in Melbourne found that despite not being underweight, EDNOS-Wt (Eating Disorder Not Otherwise Specified—Anorexia not meeting weight criteria; or Atypical-AN) patients experienced similar life-threatening complications of weight loss as patients with threshold AN; suggesting higher-weight adolescents who have lost large amounts of weight require careful medical assessment. Between 2005 and 2009, the percentage of cases of A-AN presenting to this service increased from 8 to 41% [113].

Introduction of ARFID in the *DSM-5* has had a significant impact on the number of individuals diagnosed with the newly defined disorder [114]. While the category was expanded to include adults, much of the research remains restricted to child and adolescent samples. Difficulties diagnosing ARFID stem from the diversity of clinical presentations associated with the disorder as well as uncertainty surrounding its aetiology [115, 116]. There is also evidence to suggest clinical presentations of ARFID may differ based on age, duration of illness, and weight, making diagnosis complex [117].

The impact of ARFID on children and adolescents appears to be significant. Assessment of diagnostic migration within children and adolescents (aged 8–21) presenting to an ED service in the US found ARFID to be present in 14% of individuals in a sample who were all previously diagnosed with EDNOS [114]. It is proposed that individuals with ARFID present with various anxieties relating to food intake, which are distinct from picky eating that is commonly seen in children and does not require treatment [115]. Researchers have also noted a small degree of diagnostic crossover between ARFID and AN in younger patient cohorts throughout their treatment, further adding to ambiguity around diagnosis [118]. Evidence relating to sub-typing of ARFID behaviours in a large Swiss sample indicates selective eating had the highest prevalence (26.1%), followed by food avoidance leading to inadequate intake (19.3%). Food avoidance based on a specific underlying fear was the least common in the cohort at 5% [88].

A large cohort study of childhood obesity and mental health disorders in Germany sought to develop a phone-based interview to assess and diagnose ARFID in children in the community that could be included as a module in the Eating Disorder Examination (EDE) interview (Fairburn and Cooper 1987) [119]. Schmidt et al. [120] considered their developed assessment tool to be reliable, identifying seven cases of ARFID within their study sample. However, results from the study were limited by the very small sample size, which lacked the statistical power to determine validity.

Assessment of selective eating in a cohort of pre-schoolers in the US (*n* = 917) indicated that a significant proportion of children had either moderate (18%) or severe (3%) selective eating behaviours leading to impaired psychological functioning [121]. Children with severe selective eating were also found to have comorbid depression and social anxiety, limiting their capacity to relate to others. Zucker et al. [121] argued that these children met diagnostic criteria for ARFID, suggesting a need for screening and early intervention programs to be delivered as early as preschool.

### Males

Issues relating to accurate assessment and diagnosis are also particularly apparent in male populations. *DSM* criteria have been criticised for being ‘female-centric,’ therefore, making male diagnosis more difficult [122].

Use of the EDE (Fairburn and Cooper 1987) scores which test for ‘core’ ED symptomatologies such as dietary restraint, eating concern and weight/shape concern, were found to have clinical utility in the assessment of EDs in a sample of female children and adolescents (*n* = 656) [123]. However, screening of males presenting to an inpatient ED service using the self-report EDE-Q found UFED to be the most common diagnosis within this group, suggesting that whilst males often do not meet criteria for full threshold disorders, their symptoms can be serious enough to warrant admission to inpatient ED services [124]. The gender bias of available assessment tools potentially contributes to this population being overlooked by clinicians, despite the significant distress and harm caused by their condition [124]. A further study of male and female ED patients found that despite their diagnosis, males were also less likely to receive a referral from their treating clinician to a specialist ED service [125].

A study conducted in a clinical sample of children and adolescents aged between 6 and 18 presenting to an ED service in the US, found males to have a significantly younger age of ED onset [126]. Kinasz et al. [126] also found that males were significantly more likely than females to present with a diagnosis other than AN and BN, with the most common diagnosis among males being OSFED. Consistent with Walker et al. [110], males in this sample presented with less severe ED symptomatology than females, although levels observed were still clinically significant.

Kinasz et al. [126] revealed a significant proportion of OSFED cases in males to be A-AN, aligning with the argument that AN diagnostic criteria is largely ‘female-centric’. Removal of amenorrhoea and addition of parent reported fear of fatness in the *DSM-5* resulted in a large number of cases of EDNOS in a cohort of Australian children and adolescents being reclassified as A-AN and AN; it is not clear whether these changes had an impact on the number of male AN cases diagnosed in this sample [127]. Similar conclusions regarding the increased prevalence of AN, A-AN and BN in clinical child and adolescent samples with application of DSM-5 criteria were found in a study conducted in the US by Ornstein et al. [114]. However, in a small study of males presenting to ED services in the US, applying DSM-5 criteria resulted in an increase from 36.4% of participants being diagnosed with AN to 48.5% [128].

## Discussion

Despite significant ramifications of delayed intervention, comparatively little research has explored screening, assessment, and diagnosis in EDs, perhaps excepting instrument validation for which there is a reasonable body of published work. Large population-based screening programs are feasible and can be effectively delivered online; however, these have mostly been trialled in university students [28, 40, 42, 44, 45]. These offer an opportunity to identify individuals at risk of an ED and may also identify unmet treatment need in both general populations and high-risk ED groups. There is strong evidence to suggest screening for EDs should be routinely implemented in the care of high-risk groups, including individuals with diabetes, women seeking reproductive healthcare, and youth [56, 61, 67, 121]. Evidence for the utility of screening in primary care is also indicated; nevertheless, researchers have questioned the efficacy of current screening and diagnostic instruments given the diverse populations seen in primary care, increasingly heterogenous presentation, and significant overlap of symptoms between the disorders [104, 107, 109, 118].

Online screening initiatives partially address low-detection in both the general population and high-risk groups by increasing reach and accessibility [46, 47, 129]. Yet, while the implementation of screening initiatives appears to prompt some improvement in rates of help-seeking [47, 48, 50], this review demonstrates screening alone will not address low treatment rates, with many individuals failing to seek support even after returning a positive result (likely due to a combination of factors including health system barriers, cost, and ambivalence) [48, 50, 92, 93, 130]. Thus, whether delivered online or face-to-face, it is important to link any screening program to accessible intervention, increased service capacity, and strategies to improve motivation for change.

This review also identified a significant unmet treatment need, resulting in increased healthcare costs in the longer term in a number of countries [96, 100]. This unmet treatment need stems from considerable barriers that continue to impede and delay correct identification and diagnosis of EDs. These include lack of clinician knowledge and training, concerns around stigma and taboo (both on the part of the patient and the healthcare professional), and lack of time [92, 104]. Individuals with a high BMI (regardless of quantity or rate of weight change), males, transgender/gender diverse individuals, and ethnic minorities, face additional barriers and are less likely to be flagged for the condition by a healthcare professional [67, 72, 92, 99, 125]. Further, while clinicians are more familiar with typical AN and BN presentations, there appears to be a gap in their knowledge regarding OSFED and UFED diagnoses [23, 103]. Efforts must be made to increase awareness and knowledge of core ED symptomatology and behaviours across diagnostic groups, particularly given the significant representation of atypical and subthreshold presentations amongst people with EDs [113, 131, 132]. EDs share a number of symptoms and traits, and some researchers suggest focusing on these in identification strategies to simplify referral for non-specialist clinicians [109]. This may be enhanced with increased block allocations of ED training within medical school curriculum, and funding for the provision of continuing education for healthcare professionals (including e-learning packages).

Research suggests clinicians and parents have difficulty identifying EDs in children [110, 112, 114]. Children tend to lose weight more quickly than adolescents or adults, but may have less severe symptomatology and are less likely to present with typical behaviours such as binge/purging and excessive exercise [110]. Thus, they have the potential to be overlooked, leading to a delay in diagnosis and treatment. Evidence from studies in clinical samples suggests this is particularly true for boys [111]. Clinicians also cite difficulties in identifying ARFID in children given diverse clinical presentations and physical profiles, as well as a small degree of crossover with AN in very young patients [118]. They can find the disorder difficult to diagnose and distinguish from non-ED-related picky eating [115]. ARFID was newly categorised in the *DSM-5*, broadening the diagnostic criteria from the previous ‘Feeding Disorder of Infancy or Early Childhood’ and including adults under the diagnosis. As such, the body of evidence for this disorder is limited.

The RR provides a broad review of recent peer-reviewed ED literature, attempting to summarise and align evidence to patient care and guide decision making for the field. Due to this broad-reaching and largely policy-driven intent, some limitations exist. A substantial number of studies on ED diagnosis identified in the initial RR search process commented on the appropriateness of *DSM-5* criteria and were not included in the analysis. Instrument validation studies were also not within scope. As a RR of available evidence for six ED diagnoses across each stage of the patient care pathway, broad search terms were used with the intention of capturing the breadth of published information available. This meant it was not possible to conduct an extensive search of the literature within each component of the care pathway for each disorder or for specific diagnostic phenotypes. Further, time constraints and absence of peer review did not allow for the inclusion of grey literature and unpublished data or implementation research. Therefore, it is likely some relevant studies were missed. Nevertheless, the review was able to meet its objective to identify gaps in research that may warrant further investigation.

The purpose of the RR is to inform a translation and research strategy, which necessitated a focus on evidence that could be readily applied to the care and treatment of individuals with EDs. This approach meant observational studies contributing to the understanding of EDs and related systemic or cultural issues not yet representing opportunities for intervention were excluded. Finally, the search strategy was not designed to identify interventions or treatments around sub-threshold ED states and study selection was not sensitive to qualitative research (due to sample sizing criteria) [37]. It was limited to studies conducted in countries with Western cultures and high-resource health systems. Combined, these factors may have limited the evidence found in the review.

This RR identified key gaps in the evidence base for screening, assessment, and diagnosis in the EDs, laying the groundwork for further research and possible health system remedies. Broadly, the review found that while means for broadscale screening and identification exist, they are rarely used and as a result, identification rates for EDs remain low, with minority and stigmatised groups particularly impacted. This means significant numbers of people are being under-treated or not treated at all, creating long-term health and health system impacts.

Future research and translation efforts may address mechanisms to increase clinical understanding, identification, and better care practice for all EDs, including atypical and sub-threshold presentations. This is particularly vital for high-risk, diverse and minority populations (e.g., LGBTQ+/gender diverse, ethnic minority groups), the latter of whom face poorer outcomes due to lack of access to services and delayed diagnosis. Such research should be translation focused and examined across healthcare and high-risk settings such as high-schools. Further, methods to reduce personal barriers to the pursuit of assessment and diagnosis is needed to drive early intervention in the illness group and lead to better outcomes for all.

## Conclusions

Despite increased advocacy in recent years, a majority of individuals with eating disorders remain undiagnosed and untreated, particularly males and those from diverse or minority populations. Research into improving detection and clinician diagnostic skill is extremely limited. Innovative empirical research is strongly recommended to address significant individual and health-system barriers currently preventing appropriate and timely intervention for many.

## Supplementary Information

**Additional File 1.** PRISMA diagram: Rapid Review.

**Additional File 2.** Studies included in the Rapid Review.

## Data Availability

Not applicable—all citations provided.

## Abbreviations

A-AN: Atypical Anorexia Nervosa
AN: Anorexia Nervosa
ARFID: Avoidant Restrictive Food Intake Disorder
BED: Binge Eating Disorder
BMI: Body Mass Index
BN: Bulimia Nervosa
DSM-5: Diagnostic and Statistical Manual of Mental Disorders 5
ED: Eating Disorder
EDNOS: Eating Disorder Not Otherwise Specified
FED: Feeding or Eating Disorder
HBI: Healthy Body Image program
NEDA: National Eating Disorders Association
OSFED: Other Specified Feeding or Eating Disorder
PD: Purging Disorder
RR: Rapid Review
UFED: Unspecified Feeding or Eating Disorder
