# Supplementary material for: Screening, assessment and diagnosis in the eating disorders: findings from a rapid review
Source: J Eat Disord. 2022 Jun 7;10:78. doi: 10.1186/s40337-022-00597-8 (PMC9175461; doi:10.1186/s40337-022-00597-8)
Supplement: Supplementary file 1 — Additional File 1. PRISMA diagram: Rapid Review. [file 40337_2022_597_MOESM1_ESM.docx]

**Additional File 1. PRISMA diagram – Rapid Review**

Articles identified through database searching

(n=17,757)

Articles identified through links and reference lists

(n=36)

Articles after duplicates removed

(n=9,260)

Articles screened through assessment of abstract/title

(n=9,260)

Excluded

(n=7,292)

Full text articles assessed for eligibility

(n=1,968)

Excluded

(n=660)

Expert research collaborative requested articles

(n=12)

Articles included in Rapid Evidence Review

(n=1,320)

Identification

Screening

Eligibility

Included
