# Supplementary material for: Screening, assessment and diagnosis in the eating disorders: findings from a rapid review
Source: J Eat Disord. 2022 Jun 7;10:78. doi: 10.1186/s40337-022-00597-8 (PMC9175461; doi:10.1186/s40337-022-00597-8)
Supplement: Supplementary file 2 — Additional File 2. Studies included in the Rapid Review. [file 40337_2022_597_MOESM2_ESM.docx]

**Additional File 2.** Studies included in the Rapid Review

| **Author, Year** | **Country** | ***N*** | **Population** | **Aim** | **Design** | **Outcome Measure** |
| --- | --- | --- | --- | --- | --- | --- |
| **Primary research articles** | | | | | | |
| Allen et al., 2011 | Australia | 212 | Outpatient (Adult, All Genders) | To evaluate the accuracy of eating disorder diagnoses made in primary care settings, and to compare the accuracy of primary care diagnoses with those generated using patient self-report questionnaires. | Cross-Sectional | Accuracy of ED diagnosis made in primary care settings |
| Baldofski et al., 2015 | Germany | 233 | Community (Adult, All Genders) | To investigate the prevalence of BED and NES and associations with various forms of nonnormative eating behavior and psychopathology in prebariatric patients. | Cross-Sectional | ED psychopathology and diagnosis of BED or NES using Eating Disorder Examination questionnaire |
| Bruneau et al., 2017 | France | 60 | Women seeking fertility treatment | Evaluate the prevalence of EDs in women seeking treatment for infertility, and to better characterize their clinical profile. | Cross-Sectional | ED prevalence & symptomatology |
| Burton Murray et al., 2020 | US | 288 | Patients referred for gastroparesis/dyspepsia symptoms, adults, all genders | To determine: (a) FED symptom frequency, and (b) relation of FED symptoms to gastrointestinal symptom severity and gastric retention abnormalities among patients presenting with gastroparesis/dyspepsia symptoms | Cross-Sectional | FED prevalence & relationship with gastro symptoms/severity |
| Bye et al., 2018 | UK | 134 | Study 1: pregnant and postnatal women with current or past ED, Study 2: student or professional midwives | Understand the barriers to disclosure and identification of ED in pregnancy and postnatally as perceived by women with past or current ED, and midwives and health visitors working in the UK National Health Service. | Mixed methods: survey + focus groups | Barriers to disclosure and identification of ED in pregnancy and postnatally as perceived by women with past or current ED, and midwives and health visitors working in the UK NHS. |
| Caudle et al., 2015 | Australia | 285 | Outpatient (Adult, All Genders) | To evaluate the changes in percentages of patients in a residual DSM-IV category compared to a residual DSM-5 category by retrospectively applying DSM-5 criteria to the clinical records of a patient population in a clinical setting; and to compare the psychopathology between the EDNOS and OSFED/UFED groups. | Retrospective cohort study | DSM-IV vs DSM-V diagnoses |
| Coker, Telfer & Abraham 2015 | Australia | 1038 | Obstetric, routine, gynaecological, psychiatric and ED inpatients and outpatients, females, adults | To compare prevalence of problems with body weight, eating and exercise (past or present) of female psychiatric inpatients with routine care, gynaecological and obstetric female outpatients, and eating disorder inpatients. | Cross-Sectional | ED prevalence & symptomatology |
| Costa & Pinto, 2015 | Brazil | 96 | Community (Adult, All Genders) | To evaluate the association between the presence and the level of binge eating disorder and the quality of life of the obese candidates for bariatric surgery. | Cross-Sectional | BMI, binge eating pathology and quality of life |
| Cousins et al., 2015 | US | 85 | Women seeking fertility treatment or attending routine primary care; 18-44years | To compare eating disorder (ED) symptoms in women seeking treatment for infertility to women receiving routine primary care. | Cross-Sectional | ED prevalence & symptomatology |
| da Luz et al., 2018 | Australia | 6052 | Community (Mixed Cohort, All Genders) | To investigate disordered eating behaviours and cognitions in a community representative sample of 6052 participants in South Australia, aged 15–99 years. | Cross-Sectional | ED/DE prevalence & symptomatology |
| Dahl et al., 2010 | Norway | 157 | Community (Adult, All Genders) | To study the prevalence of all relevant eating disorders in obese patients waiting for bariatric surgery and to explore for possible differences between gender and ED status. | Cross-Sectional | ED psychopathology and diagnostic status |
| Diaz et al., 2013 | Spain | 45 | Community (Adult, All Genders) | To assess whether patients with at least one binge episode per week have worse outcomes after bariatric surgery than those without BED, in terms of weight loss, resolution of comorbidities, surgical complications, quality of life and Bariatric Analysis Reporting and Outcome System (BAROS) overall score. | Longitudinal (<5yr) | Binge eating pathology, weight loss, comorbidities, surgical complications and quality of life. |
| Domine et al., 2009 | Switzerland | 3890 | Community (Young Adult, Men) | To determine the characteristics specific to boys with disordered eating behaviours (DEB) and the general context in which these DEB occur. | Cross-Sectional | Disordered eating prevalence |
| Dooley-Hash et al., 2019 | US | 1795 | Emergency department patients, all genders, adults | Describe the prevalence of eating disorders among adult patients who present to the emergency department for medical care and examines the relationship between eating disorders, depression, and substance use disorders. | Cross-Sectional | ED prevalence & relationship between ED, depression and SUD |
| Eilander et al., 2017 | Netherlands | 103 | Community (Adolescents, All Genders) | To (i) explore the prevalence of DEBs and associated ‘yellow flags’, and (ii) establish concordance between adolescents–parents and adolescents–clinicians with respect to DEBs. | Cross-Sectional | DEB prevalence |
| Eisenberg et al., 2011 | US | 2822 | College (Adult, All Genders) | To examine the prevalence, correlates, persistence, and treatment-seeking related to symptoms of eating disorders (EDs) in a random sample of college students. | Cross-Sectional | ED prevalence, correlates & treatment seeking |
| Ekeroth et al., 2013 | Sweden | 2233; 530 at follow-up | Inpatients and outpatients from national ED database | To examine clinical characteristics and distinctiveness of the new DSM-5 ED diagnoses, especially concerning purging disorder (PD). | Repeated measure (with follow-up) | Clinical characteristics and distinctiveness of the new DSM-5 ED diagnoses |
| Fitzsimmons-Craft et al., 2019 | US | 4894 | College (Adult, All Genders) | To report on: (a) reach of the Healthy Body Image program, (b) screen results, and (c) differences across ED status groups. | Cross-Sectional + CT intervention | HBI Integrated ED screening & intervention: Risk status; symptom reduction following mobile app intervention |
| Fitzsimmons-Craft, et al., 2019 | US | 71,362 | Community (Adult, All Genders) | To determine the reach of disseminating an online eating disorder screener in partnership with the National Eating Disorders Association (NEDA), as well to examine the probable eating disorder diagnostic and risk breakdown of adult respondents. We also assessed receipt of any treatment. | Cross-Sectional | Screen - risk & prevalence |
| Fitzsimmons-Craft et al., 2019 | US | 2454 | College (Adult, All Genders) | Report on (a) reach of HBI in the Missouri public universities over the course of 3 years, (b) results of the screen and differences in risk/clinical status groups on demographic variables, (c) uptake of the online/mobile interventions, and (d) results regarding the effectiveness of the mobile SB-ED intervention for students who screen positive for a clinical/subclinical ED other than AN. | Cross-Sectional (screening) + CT intervention | HBI Integrated ED screening & intervention: Risk status; symptom reduction following mobile app intervention |
| Fitzsimmons-Craft et al., 2020 | US | 61 | Community (Adult, All Genders) | To conduct a longer-term (i.e., 9-month) follow-up of students identified with possible anorexia nervosa (AN) as part of the Healthy Body Image Program, | Repeated Measure (with follow-up) | Changes in ED pathology, comorbidity and help-seeking 9-months post positive AN screen |
| Fitzsimmons-Craft et al., 2020 | US | 16,396 | NEDA online screen respondents who screen positive or high risk for an ED | To understand help-seeking among respondents to the National Eating Disorders Association online screen who screened positive or at high risk for an ED. | Repeated Measure (with follow-up) | Help-seeking intention post positive ED screen (online) |
| Flatt et al., 2021 | US | 23,920 | Community (Mixed Cohort, All Genders) | Compare eating disorder (ED) characteristics and treatment seeking behaviours between self-identified competitive athletes and non-athletes in a large, community-based sample. | Cross-Sectional | ED characteristics & treatment seeking |
| Forbush et al., 2017 | US | 229 | Community (Adult, All Genders) | Develop a transdiagnostic, hierarchical-dimensional model relevant to ED psychopathology that: 1) reduces diagnostic heterogeneity, 2) includes important dimensions of internalizing psychopathology that are often excluded from ED diagnostic models, and 3) predicts clinical impairment. | Cross-Sectional | Validity of a new transdiagnostic, hierarchical-dimensional model relevant to ED psychopathology that: 1) reduces diagnostic heterogeneity, 2) includes important dimensions of internalizing psychopathology that are often excluded from ED diagnostic models, and 3) predicts clinical impairment. |
| Freizinger 2010 | US | 82 | Women seeking fertility treatment | To determine the prevalence of eating disorders in a sample of infertile women | Cross-Sectional | ED prevalence & symptomatology |
| Fursland & Watson, 2014 | Australia | 260 | Psychiatric outpatients, all genders, adults | To identify the prevalence of eating problems, using the SCOFF, and eating disorders when screening positive on the SCOFF (i.e., ≥2), among patients seeking help for anxiety and depression at a community-based mental health service. | Cross-Sectional | ED prevalence & symptomatology |
| Gooding et al., 2016 | US | 303 | Paediatricians and nurse practitioners or assistants | Evaluate the ability of two educational interventions to increase screening for eating disorders in paediatric primary care practice | Quasi-experimental (intervention) | Documented screening of patients following ED education |
| Hartman-Munick et al., 2021 | US | 66 | Transgender young adults | To understand transgender young adult experiences with ED screening and treatment | Cohort study | Experiences with ED screening & treatment |
| Ivancic et al., 2021 | Australia | 1.57mil | Community (Mixed Cohort, All Genders) | To estimate the prevalence of management of eating disorders in primary care and identify how these disorders are managed. | Modelling (Statistical) | Prevalence and management of eating disorders in primary care |
| Ivezaj et al., 2014 | US | 97 | Community (Adult, All Genders) | To examine weight change trajectories among overweight and obese patients with binge eating disorder (BED) versus without (NBO) during the year prior to seeking treatment. | Retrospective correlation study | Weight change trajectories among overweight and obese patients with binge eating disorder (BED) versus without (NBO) during the year prior to seeking treatment. |
| Jeanes et al., 2016 | UK | 495 | College (Adult, Women) | To report the prevalence of binge eating and food cravings and their relation to obesity risk in women with PCOS. | Cross-Sectional | Prevalence of binge eating and food cravings and relation to obesity risk in women with PCOS |
| Jennings & Phillips, 2017 | US | 38 | Inpatient, all ages, males | Present normative data on the Eating Disorder Examination Questionnaire (EDE-Q) for a clinical sample of males in the United States | Cross-Sectional | Clinical male norms on EDE-Q |
| Jones et al., 2014 | US | 1551 | College (Adult, All Genders) | Describe the pilot implementation of the Healthy Body Image program at universities, using two implementation approaches: solicited screening and universal screening | Cross-Sectional + targeted prevention program | HBI Integrated ED screening & intervention: Risk status; symptom reduction following mobile app intervention |
| Kass et al., 2015. | US | 1529 | College (Adult, All Genders) | To evaluate differential eating disorder screening responses by initial weight status among university students, to assess eating disorder risk and pathology among individuals with overweight/obesity versus normal weight or underweight. | Cross-Sectional | Differential eating disorder screening responses by initial weight status |
| Kinasz et al., 2016 | US | 619 | Inpatients and outpatients, young people, all genders | To compare demographic and clinical characteristics of child and adolescent males and females who presented for ED treatment. | Cross-Sectional cohort study | ED characteristics, males vs females |
| Kurz et al., 2016 | Switzerland | 1444 | Community (Children, All Genders) | To determine the factor structure of the newly developed self-report screening questionnaire Eating Disturbances in Youth-Questionnaire (EDY-Q) as well as to report the distribution of variants of early-onset restrictive eating disturbances characteristic of avoidant/restrictive food intake disorder (ARFID) in a middle childhood population sample. | Cross-Sectional | Factor structure of EDY-Q & prevalence of early onset restrictive eating disturbances |
| Lakeman & McIntosh, 2018 | Australia | 136 | Clinicians working in emergency, paediatric and mental health wards | To identify the educational preparedness, competence and confidence of clinicians to work with people with EDs; and to identify how services might be improved. | Cross-Sectional | Educational preparedness, competence and confidence of clinicians to work with people with ED |
| Limburg et al., 2018 | Australia | 1062 | Outpatient (Young People, All Genders) | To compare the DSM-IV, DSM-5, and ICD-10 eating disorders (ED) nomenclatures to assess their value in the classification of paediatric eating disorders. We investigated the prevalence of the disorders in accordance with each system's diagnostic criteria, diagnostic concordance between the systems, and interrater reliability. | Cross-Sectional | Prevalence of ED per DSM-IV, DSM-5, and ICD-10 eating disorders (ED) nomenclature- to assess their value in the classification of paediatric eating disorders |
| Lipson et al., 2017 | US | 2180 | College (Adult, All Genders) | To report findings from a two-phase pilot study designed to understand: (1) why students with significant untreated ED symptoms do not seek help (i.e., to identify salient treatment barriers), and (2) engagement in universal intervention and prevention programs. | Cross-Sectional + targeted prevention program | HBI Integrated ED screening & intervention: Risk status; symptom reduction following mobile app intervention |
| Lobera et al., 2009 | Spain | 93 | Psychiatric outpatients, all genders, adults | a) To analyse the prevalence of eating disorders (ED) in patients referred from primary care for psychiatric assessment; and b) to analyse the psychopathological variables associated with these disorders. | Cross-Sectional | ED prevalence & symptomatology |
| Lowe et al., 2019 | US | 102 | Inpatients and outpatients, young people, all genders | To examine differences among youth with avoidant/restrictive food intake disorder (ARFID) by age, weight status, and symptom duration. A secondary goal was to report the frequencies of ARFID using DSM-5 clinical presentations | Cross-Sectional | Clinical & demographic characteristics of ARFID presentations |
| Lydecker, Shea & Grilo, 2018 | US | 2017 | Community (Adult, All Genders) | To compare eating-disorder psychopathology, frequency of purging behaviours, depression, and physical activity among individuals who reported regular methods of purging/compensatory behaviours. | Cross-Sectional | Eating-disorder psychopathology, frequency of purging behaviours, depression, and physical activity among individuals who report regular methods of purging/compensatory behaviours. |
| MacCaughelty, Wagner & Rufino, 2016 | US | 136 | Inpatient (Adult, All Genders) | To examine whether sex, age, body mass index (BMI), and eating disorder diagnosis were associated with referral rates for eating disorder consults in a general inpatient psychiatric facility. | Cross-Sectional | Association between sex, age, body mass index (BMI), and eating disorder diagnosis with referral rates for eating disorder consults |
| Maguen et al., 2017 | US | 407 | Female veterans | To develop a primary care eating disorder screen with greater accuracy and greater potential for generalizability, compared to existing screens. | Cross-Sectional | ED prevalence & Screen for Disordered Eating discriminative accuracy |
| Mitchell et al., 2015 | US | 2266 | Community (Adult, All Genders) | To describe eating patterns, prevalence of problematic eating behaviors, and determine factors associated with binge eating disorder (BED), before bariatric surgery. | Cross-Sectional | ED psychopathology, ED diagnosis, treatment history, depressive symptoms, interpersonal support and quality of life. |
| Mond & Arrighi, 2011 | Australia | 402 | Community (Adult, All Genders) | To examine gender differences in perceptions of the severity and prevalence of AN and BN among young men and women. | Cross-Sectional | Perception of prevalence, severity and associated impairment of AN and BN |
| Norris et al., 2014 | Canada | 205 | Inpatients and outpatients, young people, all genders | To assess and compare clinical characteristics of patients with avoidant/restrictive food intake disorder (ARFID) to those with anorexia nervosa (AN). | Retrospective chart review | Clinical characteristics of patients with ARFID vs AN |
| O'Brien et al., 2014 | Australia | 665 | Outpatient (Young People, All Genders) | To test the factor structure of the eating disorder examination (EDE) in a clinical paediatric sample. | Cross-Sectional | Factor structure of EDE in a clinical paediatric sample |
| Ornstein et al., | US | 215 | Outpatient (Young People, All Genders) | To determine the distribution of eating disorders (ED) in children and adolescents comparing the fourth edition of the Diagnostic and Statistical Manual (DSM) to the proposed fifth edition DSM criteria. | Cross-Sectional | DSM-IV vs DSM-V diagnoses |
| Preti et al., 2009 | Europe | 4139 | Community (Adult, All Genders) | Investigate the prevalence of non-psychotic mental disorders in Belgium, France, Germany, Italy, the Netherlands and Spain | Epidemiological/population study | ED prevalence |
| Rodino, Byrne & Sanders, 2016 | Australia | 385 | Women seeking fertility treatment | To gauge the prevalence of eating disorders in women attending a fertility clinic and to compare current disordered eating attitudes and exercise amongst different infertility categories. | Cross-Sectional | ED prevalence & symptomatology |
| Rodino, Byrne & Sanders, 2017 | Australia | 80 | Fertility specialists | To gauge fertility specialists’ knowledge, clinical practices, and training needs in regard to eating disorders. | Cross-Sectional | ED knowledge, clinical practices, and training needs. |
| Santos et al., 2017 | Brazil | 913 | Pregnant women | To determine the presence of eating disorders and its association with anxiety and depression symptomatology in high-risk pregnancies. | Cross-Sectional | ED prevalence & association with anxiety/depression in high-risk pregnancies |
| Schmidt et al., 2019 | Germany | 39 | Community (Children, All Genders) | Develop and validate an ARFID module for the child and parent version of the Eating Disorder Examination (EDE) in a nonclinical sample | Cross-Sectional | Presence and clinical characteristics of ARFID in a non-clinical sample; validation of an ARFID module for parent EDE |
| Silen et al., | Finland | 1347 | Twins, adults, all genders | Assess the detection, treatment and outcomes of DSM-5 eating disorders in a nationwide community setting. | Cross-Sectional twin study | Detection, treatment and outcomes of DSM-5 eating disorders in a nationwide community setting. |
| Simioni & Cottencin, 2016 | France | 203 | Smokers seeking cessation treatment, all genders, adults | To estimate (1) the feasibility and pertinence of implementing systematic screening for eating disorders (EDs) in outpatient smoking cessation (SC), and (2) the acceptance of a referral to ED-specific treatment. | Cross-Sectional | ED prevalence & screening feasibility |
| Swenne 2016 | Sweden | 275 | Inpatient (Young People, Women) | To study the influence of premorbid BMI on clinical characteristics of adolescent girls presenting with an ED | Retrospective correlation study | Influence of premorbid BMI on clinical characteristics of adolescent girls presenting with an ED |
| Tavolacci et al., 2015 | France | 3457 | College (Adult, All Genders) | To determine the prevalence of eating disorders among university students and its relationship to behavioural characteristics and substance use. | Cross-Sectional | ED prevalence & relationship to behavioural characteristics/substance abuse |
| Tay et al., 2018 | Australia | 8467 | Community (Adult, Women) | To evaluate the prevalence of eating disorders in women with polycystic ovary syndrome (PCOS) compared with women without PCOS and examine the relationship between PCOS, body mass index, self-esteem, and psychological distress scores. | Cross-Sectional | ED prevalence & relationship between PCOS, BMI, self-esteem and psychological distress. |
| Tenconi et al., 2015 | Italy | 150 | Outpatient (Adult, All Genders) | To provide data about the role of obstetric complications (OCs) in a large and well-characterized sample of patients with anorexia nervosa (AN) or bulimia nervosa (BN). | Cross-Sectional | History of obstetric complications in individuals with ED |
| Utzinger et al., 2016 | US | 242 | Community (Adolescents, All Genders) | To assess loss of control (LOC) over eating and eating disorders (EDs) in adolescents undergoing bariatric surgery for severe obesity. | Cross-Sectional | ED psychopathology, night eating, weight patterns, depressive symptoms and quality of life. |
| Valente et al., 2017 | Italy | 267 | Outpatient (Adult, All Genders) | To highlight the characteristics of eating disorders (ED) in males, with particular attention to sex-related clinical features and psychiatric co-morbidities. | Cross-Sectional | Characteristics of eating disorders (ED) in males |
| Vo, Lau & Rubinstein., 2018 | US | 33 | Outpatient (Young People, Men) | To describe the demographic characteristics, presenting vital signs, laboratory results, and relevant risk factors for eating disorders among males presenting to an outpatient adolescent and young adult medicine practice. | Retrospective chart review | Clinical & demographic characteristics of males presenting with ED |
| Wade & O'Shea, 2015 | Australia | 699 | Community (Adolescents, Girls) | To explore what disorders in UFED might look like in an adolescent population. | Repeated measure (without follow-up) | UFED presentation & characteristics |
| Walker et al., 2014 | Australia | 656 | Outpatient (Young People, All Genders) | To examine child and adolescent differences in the clinical presentation of eating disorders (EDs) at referral to a specialist paediatric program. | Cross-Sectional | Characteristics of eating disorders in children vs adolescents |
| Waller et al., 2009 | UK | 648 | Inpatients and outpatients, all ages, all genders | This study considered the impact of ethnicity on the referral process for patients with eating disorders, at the levels of referral rate, diagnosis, and treatment offered. | Cross-Sectional cohort study | Impact of ethnicity on the referral process for patients with eating disorders |
| Webb, Applegate & Grant, 2011 | US | 488 | Community (Adult, All Genders) | To identify socio-demographic and psychological correlates of the co-occurrence of Type 2 DM and BED among bariatric surgery candidates. | Cross-Sectional | BMI, depressive symptoms, hedonic hunger/food locus of control beliefs, severity of binge eating-related cognitions |
| Whitelaw et al., 2014 | Australia | 99 | Inpatient (Young People, All Genders) | To describe the changing incidence of EDNOS-Wt compared with AN, and to compare the characteristics of these 2 groups in a cohort that required hospitalization after weight loss. | Restrospective cohort study | Clinical characteristics of AN vs EDNOS-Wt (or Atypical AN) |
| Ziobrowski et al., 2019 | US | 3649 | Community (Mixed Cohort, Girls) | To assess whether girls with mothers who have had an eating disorder (ED) have greater odds of developing ED symptoms and whether girls with ED symptoms have greater odds of receiving ED treatment if their mothers have an ED history. | Repeated Measure (without follow-up) | Correlation between maternal ED history and daughter developing ED and/or receiving treatment for ED |
| Zucker et al., 2015 | US | 531 | Community (Children, All Genders) | Examine the clinical significance of moderate and severe selective eating (SE). | Repeated Measure (with follow-up) | Clinical significance of moderate and severe selective eating (SE) |
| **Review articles** | | | | | | |
| Amianto et al., 2015 | Italy | - | N/A | To explore the available data on this topic, outlining the state-of-the-art on both diagnostic issues and most effective treatment strategies. | Review (Systematic) | N/A |
| Bode et al., 2017 | Germany | - | N/A | To conduct cost-offset analyses for evidence-based treatment of eating disorders using outcome data from a psychotherapy trial involving cognitive behavioral therapy (CBT) and focal psychodynamic therapy (FPT) for AN and a trial involving CBT for BN. | Review (Other) | Cost-offset (reduction in direct healthcare costs, reduction in indirect costs, annual treatment costs, QALYs) |
| Bourne et al., 2020 | UK | - | N/A | To systematically assess the extent and nature of the ARFID literature, to identify gaps in current understanding, and to make recommendations for further study. | Review (Systematic) | The extent and nature of ARFID literature |
| Bringham et al., 2018 | US | - | N/A | To evaluate the identification and treatment of ARFID in adolescents | Review (Narrative) | N/A |
| Cadwaller et al., 2016 | France | - | N/A | To describe the effect (clinical outcomes and care trajectory) of screening for EDs among patients in general practice settings. | Review (Systematic) | Effect (clinical outcomes and care trajectory) of screening for EDs among patients in general practice settings. |
| De Zwann, Marshchollek & Allison, 2015 | Worldwide | - | N/A | To review the literature on night eating syndrome. | Review (Critical) | Circadian rhythm, obesity, bariatric surgery and treatment of NES |
| Hanlan et al., 2015 | US | - | N/A | To review the prevalence of eating disorders and disordered eating behaviours in individuals with type 1 diabetes | Review (Narrative) | N/A |
| Hart et al., 2011 | Australia | - | N/A | To systematically review the literature on the proportion of community cases with a diagnosable eating disorder who seek eating disorder specific treatment. | Review (Systematic) | N/A |
| Kornstein et al., 2016 | US | - | N/A | Review the clinical skills needed to recognize, diagnose and manage BED in a primary-care setting | Review (Systematic) | N/A |
| Lee et al., 2018 | US | - | N/A | To identify the risk of eating disorders (ED) in women with polycystic ovary syndrome (PCOS) compared to controls. | Systematic Review/ Meta-Analysis (combined) | N/A |
| Murray, S B, et al. 2017 | US/Australia | - | N/A | Provide an overview of the history of male EDs and synthesizes current evidence relating to the unique characteristics of male presentations across the diagnostic spectrum of disordered eating. | Review (Critical) | N/A |
| Nazar et al., 2017 | UK | - | N/A | A systematic review of the literature and meta-analyses examined the robustness of this concept. | Systematic Review/ Meta-Analysis (combined) | N/A |
| Opolski, Chur-Hansen & Wittert 2015 | Worldwide | - |  | \| To provide a critical evaluation of current literature on eating-related issues  in pre-surgical bariatric candidates. \| \| --- \| \|  \| | Review (Systematic) | Presence of eating disorder diagnosis, grazing, emotional eating, food cravings and food addiction among bariatric candidates. |
| Paganini et al., 2018 | Australia | - | N/A | To investigate the relationship between binge eating, in the broader context of eating disorder behaviours, and Polycystic Ovarian Syndrome (PCOS), taking into account shared characteristics between EDs (Eating Disorders) and PCOS. | Review (Systematic) | N/A |
| Strand, Hausswolff-Juhlin & Welch, 2018 | Sweden | - | N/A | Explore how ARFID as a diagnostic entity is conceptualized in the research literature and evaluates the diagnostic validity according to the Feighner criteria. | Review (Systematic) | N/A |
| Striegel Weissman & Rosselli, 2017 | US | - | N/A | Provide an update of the research literature published since 2011 in three research areas: unmet treatment needs, cost of illness, and cost-effectiveness of treatments. | Review (Narrative) | N/A |
| Toni et al., 2017 | Italy | - | N/A | To review present knowledge about the clinical relevance of EDs and DE and possible preventive and therapeutic measures used to reduce their impact and course of type 1 diabetes. | Review (Narrative) | N/A |

**Abbreviations –** AN: Anorexia Nervosa; ARFID: Avoidant Restrictive Food Intake Disorder; BED: Binge Eating Disorder; BMI: Body Mass Index; BN: Bulimia Nervosa; CT: Controlled Trial; DE: Disordered Eating; DEB: Disordered Eating Behaviour; DM: Diabetes Mellitus; DSM: Diagnostic and Statistical Manual of Mental Disorders (American Psychiatric Association); ED: Eating Disorder; EDE-Q: Eating Disorder Examination Questionnaire; EDNOS: Eating Disorder Not Otherwise Specified; EDNOS-Wt: EDNOS due to not meeting weight criteria; EDY-Q: Eating Disturbances in Youth Questionnaire; FED: Feeding or Eating Disorder; HBI: Healthy Body Image program; ICD: International Classification of Diseases manual; NBO: ‘without Binge Eating Disorder’; NEDA: National Eating Disorders Association; NHS: National Health Service; OC: Obstetric Complications; OSFED: Other Specified Feeding or Eating Disorders; PCOS: Polycystic Ovary Syndrome; PD: Purging Disorder; RCT: Randomised Controlled Trial; SB-ED: xxx; SCOFF: Sick, Control, One-stone, Fat Food questionnaire; SE: Selective Eating; SUD: Substance Use Disorder; UK: United Kingdom; UFED: Unspecified Feeding or Eating Disorder; US: United States.
